# In Memoriam—J. P. Dake, M. D.

**Published:** 1894-12

**Authors:** J. Richey Horner, E. H. Pond

**Affiliations:** Chairman Allegheny County Hom. Med. Society; Secretary


### IN MEMORIAM—J. P. DAKE, M. D.

The Homœopathic Medical Society of Allegheny County, Pa., having learned of the demise of Dr. J. P. Dake, formerly of this city (Pittsburg), and an honorary member of this Society, appointed the following Committee to prepare a suitable memorial bearing upon the sad event, viz. : J. F. Cooper, M. D., J. C. Burgher, M. D., and J. H. McClelland, M. D.

The Committee charged with this duty presented the following report at a special Memorial Meeting, held November 13th, 1894, which was unanimously adopted :

MEMORIAL UPON THE DEATH OF J. P. DAKE, A. M., M. D.

We are called upon to express our appreciation and regard for a distinguished colleague and honorary member of this Society.

Dr. J. P. Dake, formerly of Pittsburg, died after a brief illness at his home in Nashville, Tenn., upon the 28th day of October, 1894, in the sixty-seventh year of his age.

We recognize a peculiar fitness in placing upon the records of this Society a formal expression of regard for one who formerly lived in our midst, respected as a citizen, eminent in his profession, and greatly beloved by his friends.

Dr. Dake was, for a time, a pupil of the pioneer of Homœopathy west of the Alleghenies, Dr. Gustavus Richelm, and was afterward (1851) associated with him in practice in this city. His talents soon won for him first place in this community, and by the end of a decade he was so overwhelmed with professional duties that his health began to give away under the strain. In the year 1863 he was compelled to retire to his farm in Ohio, where, in a few years, he quite regained his former health and vigor. Consideration for the health of his wife determined his removal to Nashville, in which city he fulfilled a useful life and closed a singularly brilliant career.

Dr. Dake was a man of rare cultivation and refined tastes. He was none the less a man of large intellectual powers and an unceasing laborer in the various lines of literary and professional work.

As editor, author, and professor, he was alike distinguished for signal ability. In our national body, the American Institute of Homœopathy, he was a leader of acknowledged power, and the transactions for a third of a century have been enriched by his word and thought.

In this Society, and in this community, he will ever be held in highest esteem as one who dignified his calling, holding aloft the banner of medical reform, when it took courage to espouse the cause of Homœopathy, and leaving to former friends, patients, and colleagues a memory full of respect and personal regard.

J. F. Cooper, M. D.,
J. C. Burgher, M. D., } *Committee.*
J. H. McClelland, M. D.,

Eulogistic remarks were made by Dr. J. C. Burgher, a former partner of Dr. Dake, Dr. J. H. McClelland, one of his students, Dr. J. F. Cooper, who was his colleague, and Drs. C. F. Bingaman, W. J. Martin, J. B. and R. W. McClelland and L. H. Willard, who all testified to the sterling worth of the man whose loss we are called upon to mourn.

J. Richey Horner, M. D.,
*Chairman Allegheny County Hom. Med. Society.*

E. H. Pond, M. D.,
*Secretary.*

